# Inflammatory Mediators in Tracheal Aspirates of Preterm Infants Participating in a Randomized Trial of Permissive Hypercapnia

**DOI:** 10.3389/fped.2017.00246

**Published:** 2017-11-21

**Authors:** Sarah Gentner, Mandy Laube, Ulrike Uhlig, Yang Yang, Hans W. Fuchs, Jens Dreyhaupt, Helmut D. Hummler, Stefan Uhlig, Ulrich H. Thome

**Affiliations:** ^1^Division of Vascular Surgery, University of Ulm, Ulm, Germany; ^2^Center for Pediatric Research Leipzig, Hospital for Children and Adolescents, Division of Neonatology, University of Leipzig, Leipzig, Germany; ^3^Institute of Pharmacology and Toxicology, RWTH Aachen University, Aachen, Germany; ^4^Center for Pediatrics, Medical Center – University of Freiburg, Faculty of Medicine, University of Freiburg, Freiburg, Germany; ^5^Institute of Epidemiology and Medical Biometry, University of Ulm, Ulm, Germany; ^6^Division of Neonatology and Pediatric Critical Care, Department of Pediatrics, University of Ulm, Ulm, Germany

**Keywords:** permissive hypercapnia, bronchopulmonary dysplasia, pulmonary inflammation, tracheal aspirates, preterm infants

## Abstract

**Background:**

Ventilator-induced lung injury is considered to be a main factor in the pathogenesis of bronchopulmonary dysplasia (BPD). Optimizing ventilator strategies may reduce respiratory morbidities in preterm infants. Permissive hypercapnia has been suggested to attenuate lung injury. We aimed to determine if a higher PCO_2_ target range results in less lung injury compared to the control target range and possibly reduces pro-inflammatory cytokines and acid sphingomyelinase (ASM) in tracheal aspirates (TA), which has not been addressed before.

**Methods:**

During a multicenter trial of permissive hypercapnia in extremely low birthweight infants (PHELBI), preterm infants (birthweight 400–1,000 g, gestational age 23 0/7–28 6/7 weeks) requiring mechanical ventilation within 24 h of birth were randomly assigned to a high PCO_2_ target or a control group. The high target group aimed at PCO_2_ values of 55–65, 60–70, and 65–75 mmHg and the control group at PCO_2_ values of 40–50, 45–55 and 50–60 mmHg on postnatal days 1–3, 4–6, and 7–14, respectively. TA was analyzed for pro-inflammatory cytokines from postnatal day 2–21. BPD was determined at a postmenstrual age of 36 weeks ± 2 days.

**Main findings:**

Levels of inflammatory cytokines and ASM were similar in both groups: interleukin (IL)-6 (*p* = 0.14), IL-8 (*p* = 0.43), IL-10 (*p* = 0.24), IL-1β (*p* = 0.11), macrophage inflammatory protein 1α (*p* = 0.44), albumin (*p* = 0.41), neuropeptide Y (*p* = 0.52), leukotriene B_4_ (*p* = 0.11), transforming growth factor-β_1_ (*p* = 0.68), nitrite (*p* = 0.15), and ASM (*p* = 0.94). Furthermore, most inflammatory mediators were strongly affected by the age of the infants and increased from postnatal day 2 to 21. BPD or death was observed in 14 out of 62 infants, who were distributed evenly between both groups.

**Conclusion:**

The results suggest that high PCO_2_ target levels did not result in lower pulmonary inflammatory activity and thus reflect clinical results. This indicates that high PCO_2_ target ranges are not effective in reducing ventilator-induced lung injury in preterm infants, as compared to control targets.

**Trial registration:**

ISRCTN56143743.

## Introduction

Bronchopulmonary dysplasia (BPD), a form of chronic lung disease, is frequently observed in preterm infants. BPD development is associated with long-term oxygen supplementation (1, 2), and frequent re-admissions to hospitals (3, 4), resulting in high health-care costs (5). Lung damage and developmental arrest induced by BPD are mainly irreversible and the respiratory impairment may continue into adolescence and adulthood (6, 7).

Ventilator-induced baro/volutrauma represents one of the main factors in the pathogenesis of lung injury and subsequent BPD development and is mainly related to the magnitude of tidal volumes (8, 9). Permissive hypercapnia is a therapeutic strategy that attempts to minimize baro/volutrauma by reducing tidal volumes, which may result in alveolar hypoventilation with increased blood partial pressure of carbon dioxide (PCO_2_). Possible benefits of permissive hypercapnia such as diminished lung injury and pulmonary inflammation (10) might be due to the reduction of lung stretch that occurs when tidal volumes are minimized. Some retrospective analyses suggested that higher arterial PCO_2_ values in the first days of life of preterm infants might be associated with a reduced incidence of BPD (11, 12), whereas other studies did not (13, 14). Notably, several previous randomized trials of permissive hypercapnia did not demonstrate a reduction of BPD incidence (15–17), which might be due to small PCO_2_ differences between groups or limited sample sizes.

The preterm lung lacks antioxidant capacity and anti-inflammatory mediators, leading to enhanced oxygen toxicity, inflammatory reactions, and repair processes (18–20). Thus, control of inflammatory processes is disturbed in immature lungs and inflammatory reactions may be prolonged and more damaging than in the mature lung. Cytokines can be measured in tracheal aspirates (TA) and reflect the extent of the inflammatory reactions (21, 22). Elevated levels of interleukin (IL)-1, IL-6, IL-8, intercellular adhesion molecule-1, macrophage inflammatory protein (MIP)-1α, transforming growth factor (TGF)-β_1_, and leukotriene B_4_ (LTB_4_) were detected within the first 10 days of life in the bronchoalveolar lavage fluid of preterm infants who subsequently developed BPD compared to infants who did not (23–29). In multicenter trials of high-frequency oscillatory ventilation (30) and inhaled nitric oxide (31), the clinical outcome was predicted by the IL-8 and LTB_4_ TA levels (32, 33). Furthermore, increased glycolipids, such as ceramide, were detected in the bronchoalveolar lavage fluid of patients with acute respiratory distress syndrome (34) and ceramide has been shown to induce apoptosis in lung epithelial cells (35, 36). More recently, ceramide and acid sphingomyelinase (ASM), the enzyme synthesizing ceramide, were shown to be involved in edema formation in models of acute lung injury (37, 38), and ASM levels were elevated in an ovine BPD model (39). Nitrite and nitrate are breakdown products of peroxynitrite, which may be formed in inflammatory processes from superoxide and nitric oxide. Furthermore, nitrotyrosine may be formed from peroxynitrite reacting with tyrosine residues (40, 41).

Thus, in addition to inflammatory cytokines, nitrite and nitrate as well as ASM might be involved in BPD development.

During a multicenter trial of permissive hypercapnia in extremely low birthweight infants (PHELBI) (42), two different target ranges of PCO_2_ were randomly allocated in order to determine whether a higher PCO_2_ target range prevents BPD. Within this trial, TAs were collected at one study center to determine the effects of permissive hypercapnia on pulmonary inflammation. We hypothesized that permissive hypercapnia reduces pro-inflammatory cytokines and ASM in TA of preterm infants.

## Materials and Methods

### The PHELBI Trial

In brief, infants with a birthweight of 400–1,000 g and a gestational age between 23 0/7 and 28 6/7 weeks requiring endotracheal intubation and mechanical ventilation within 24 h of birth were enrolled. Infants were randomly allocated within 12 h of intubation to two different target ranges of PCO_2_. The high target group aimed at PCO_2_ values of 55–65 mmHg on postnatal days (PD) 1–3, followed by 60–70 mmHg on PD 4–6, and 65–75 mmHg on PD 7–14. The control target group aimed at PCO_2_ values of 40–50 mmHg on PD 1–3, followed by 45–55 mmHg on PD 4–6, and 50–60 mmHg on PD 7–14 (42), representing a mild hypercapnia. Thereby, the intervention aimed at a difference of 15 mmHg between the control and high target group for 14 days. Blood PCO_2_ was measured in 12-h intervals, or more frequently, if clinically indicated or when measurement results outside the target range occurred. To minimize volutrauma, a high ventilation rate (60–80/min) was favored over high tidal volumes in both groups. Initial ventilator settings comprised a rate of 60–80/min or greater, inspiratory time of 0.25–0.35 s, positive end-expiratory pressure 3.6 mbar, and a peak inspiratory pressure (PIP) resulting in minimal to moderate chest rise. The rate was allowed to decrease only if the PIP was 14 mbar or lower. Synchronized ventilation or forms of volume control were applied at the discretion of the clinicians in charge of patient care (42). TAs were sampled from infants enrolled at the largest of the study centers (Ulm, Germany).

### Tracheal Aspirate Sampling

Tracheal aspirates were sampled during normal medically indicated endotracheal suctioning procedures on PD 2, PD 4, PD 7, PD 14, and PD 21, unless the infant was extubated earlier. If less than four specimens per day were obtained, further specimen were collected on the following day as available. No TA sampling was done within 4 h of a surfactant instillation. For sampling, a standardized procedure was conducted. A sterile mucus trap was inserted in the suctioning system, followed by endotracheal instillation of 0.5 ml/kg normal saline, and brief reconnection of the ventilator (3–5 breaths). Thereafter, suctioning was performed and TA collected. Afterward, the suctioning catheter was flushed with 0.5 ml normal saline. TA were transferred to an appropriate tube and immediately centrifuged at 140 × *g* and 4°C for 10 min, whereupon, the supernatant was collected and immediately frozen. TA samples were held at −80°C until ready for shipment to the laboratory, which was done on dry ice. The number of infants from whom TA were collected declined with advancing postnatal age. Main factors for the declining number of samples were extubations within the first 21 days of life, reduced number of clinically indicated tracheal suctioning procedures due to improved pulmonary function, transfer to other hospitals, and death. All procedures of this study were approved by the institutional review board of the University of Ulm and informed parental permission was obtained.

### Tracheal Aspirate Analyses

Tracheal aspirate analyses were performed at the Institute of Pharmacology and Toxicology of the Technical University, Aachen, Germany. To determine the TA levels of IL-6, IL-8, IL-1β, IL-10, and MIP-1α, a Bio-Plex Cytokine assay (Bio-Rad Laboratories, Munich, Germany) was used (43). Enzyme-linked immunosorbent assays were conducted to analyze the TA concentrations of TGF-β_1_ (R&D Systems GmbH, Wiesbaden-Nordenstadt, Germany), albumin (AssayPro, St. Charles, IL, USA), and nitrotyrosine (Cell Sciences, Canton, OH, USA). In addition, competitive binding assays were used for neuropeptide Y (NPY) (Phoenix Europe GmbH, Karlsruhe, Germany) and LTB_4_ (R&D Systems GmbH). Nitrite TA levels were determined by a Griess reaction assay (R&D Systems GmbH). All assays were performed according to the manufacturer’s recommendations. For ASM, a proprietary assay was used as described before (44). To increase the sample amount and decrease variations of dilution, specimen from the same patient and day were pooled. To date, no uniformly accepted standard is available and thus no attempt to normalize TA levels was made. As recommended by the European Respiratory Task Force on Bronchoalveolar Lavage in children (45, 46), we expressed the data per milliliter of TA.

### Clinical Outcome

The primary outcome of the trial was death or BPD before 36 weeks postmenstrual age according to the physiological definition of BPD—i.e., requiring mechanical pressure support or supplemental oxygen at 36 weeks postmenstrual age within ±2 days, including an oxygen reduction test for infants requiring less than 0.3 FiO_2_ (BPD or death) (42, 47).

### Statistical Analyses

Demographic and clinical outcome data were compared between the high target and the control target group by Mann–Whitney *U*-test or Fisher’s exact test as appropriate. TA concentrations were compared by mixed model two-way (factors being time and target group) analyses of variance (ANOVA) with a heterogeneous unstructured covariance structure SAS software 9.4 (MIXED procedure, SAS, Cary, NC, USA). In the figures, the effect of postnatal age (time) is denoted below the *x*-axis and the target group effects on each single day at the respective time points.

## Results

During the enrollment period from 2008 to 2012, TAs were collected from 62 infants being allocated equally to the high PCO_2_ target and the control target group. Demographic data were similar between both groups with respect to the number of patients, gestational age, gender, birthweight, prenatal steroids, and infant age at intubation (Table 1).

**Table 1:** Demographic and outcome characteristics of the infants.

	Control target group	High target group	*p*-Value
Number of patients	31	31	
Gestational age (days)^a^	178 (61–199)	179 (163–201)	0.32
Birth weight (g)^a^	690 (415–970)	730 (440–990)	0.62
Male	14 (45%)	17 (55%)	0.61
Prenatal steroids	26 (84%)	29 (94%)	0.43
Apgar score 5-min^a^	8.0 (5.0–10.0)	8.5 (3.0–10.0)	0.11
Apgar score 10-min^a^	9.0 (6.0–10.0)	9.5 (5.0–10.0)	0.18
Age at intubation (h)^a^	2 (0–21)	0 (0–17)	0.54
Bronchopulmonary dysplasia^b^	5 (17%)	7 (23%)	0.75
Death	1 (3%)	0 (0%)	1.00
Intraventricular hemorrhage (all grades)^b^	12 (40%)	12 (39%)	1.00
Extubated at 36 weeks/month^b^	30 (100%)	31 (100%)	1.00
Age at extubation (days)^a^	25.5 (3–74)	20 (2–77)	0.42
No continuous positive airway pressure (CPAP) 36 weeks/month^b^	27 (90%)	26 (84%)	0.71
Age at CPAP termination (days)^a^	57 (7–79)	47 (7–73)	0.15
Mechanical ventilation (h)^a^	1,252.5 (152–2,045)	1,130 (42–2,059)	0.23
No O_2_ 36 weeks/month^b^	25 (83%)	25 (81%)	1.00

*^a^Median (minimum–maximum), Mann–Whitney *U*-test; all others: Fisher’s exact test*.

*^b^*n* (% of surviving infants)*.

**Figure 1:**
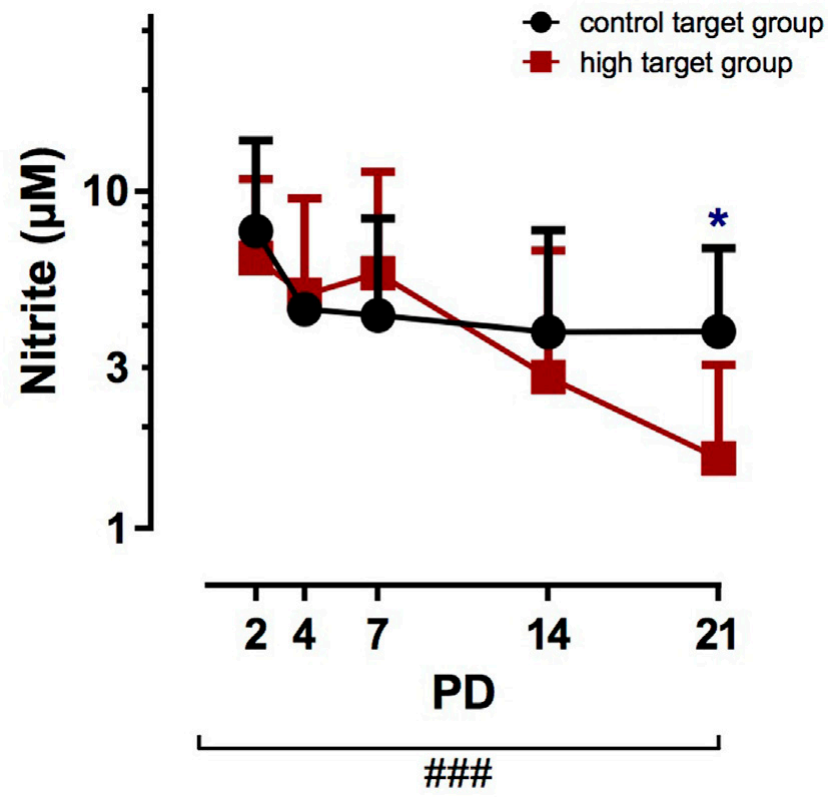
Nitrite tracheal aspirates (TA) concentration in infants treated with high PCO_2_ target levels compared to control target levels. Serial TA samples were obtained from PD2 to PD21. Number (*n*) of analyzed TA for each postnatal day and group (high PCO_2_ target group/control target group): PD2 *n* = 27/25, PD4 *n* = 24/28, PD7 *n* = 14/17, PD14 *n* = 11/15, PD21 *n* = 11/11. Data are displayed as the mean of TA levels and SD on a logarithmic scale. Nitrite TA levels decreased with advancing postnatal age in both groups (^###^*p* < 0.001). On PD2, PD4, PD7, and PD14, nitrite TA levels were not significantly affected by high PCO_2_ target levels compared to mild hypercapnia, but on PD21 the high target group demonstrated lower nitrite TA levels (^↔^*p* < 0.05). PD, postnatal day.

Mixed model ANOVA of target group and postnatal age yielded the *p*-values shown in Table 2. Furthermore, the influence of PCO_2_ was determined for each measured time point separately from PD 2 to PD 21. Nitrotyrosine TA levels were below the limit of detection (data not shown). Nitrite TA levels were similar between infants assigned to the high target and control target groups from PD 2 to PD 14 (Figure 1). On PD 21, the high target group showed significantly lower nitrite TA levels with 1.61 ± 1.45 µM (mean ± SD) compared to 3.84 ± 2.95 µM in the control group (*p* < 0.05). Furthermore, nitrite TA levels markedly decreased in both groups over the study period from PD 2 to PD 21 (*p* < 0.001).

**Table 2:** Mixed model analyses of variance (*p*-values).

	Target group	Postnatal age
Nitrite	*p* = 0.1504	*p* < 0.0001
Interleukin (IL)-6	*p* = 0.1436	*p* = 0.0382
IL-1β	*p* = 0.1075	*p* < 0.0001
Transforming growth factor-β_1_	*p* = 0.6798	*p* = 0.0007
IL-10	*p* = 0.2414	*p* = 0.0788
IL-8	*p* = 0.4268	*p* = 0.1318
Macrophage inflammatory protein-1α	*p* = 0.4445	*p* = 0.0008
Leukotriene B_4_	*p* = 0.1067	*p* = 0.0036
Neuropeptide Y	*p* = 0.5218	*p* = 0.0486
Acid sphingomyelinase	*p* = 0.9408	*p* = 0.0011
Albumin	*p* = 0.4095	*p* = 0.8114

**Figure 2:**
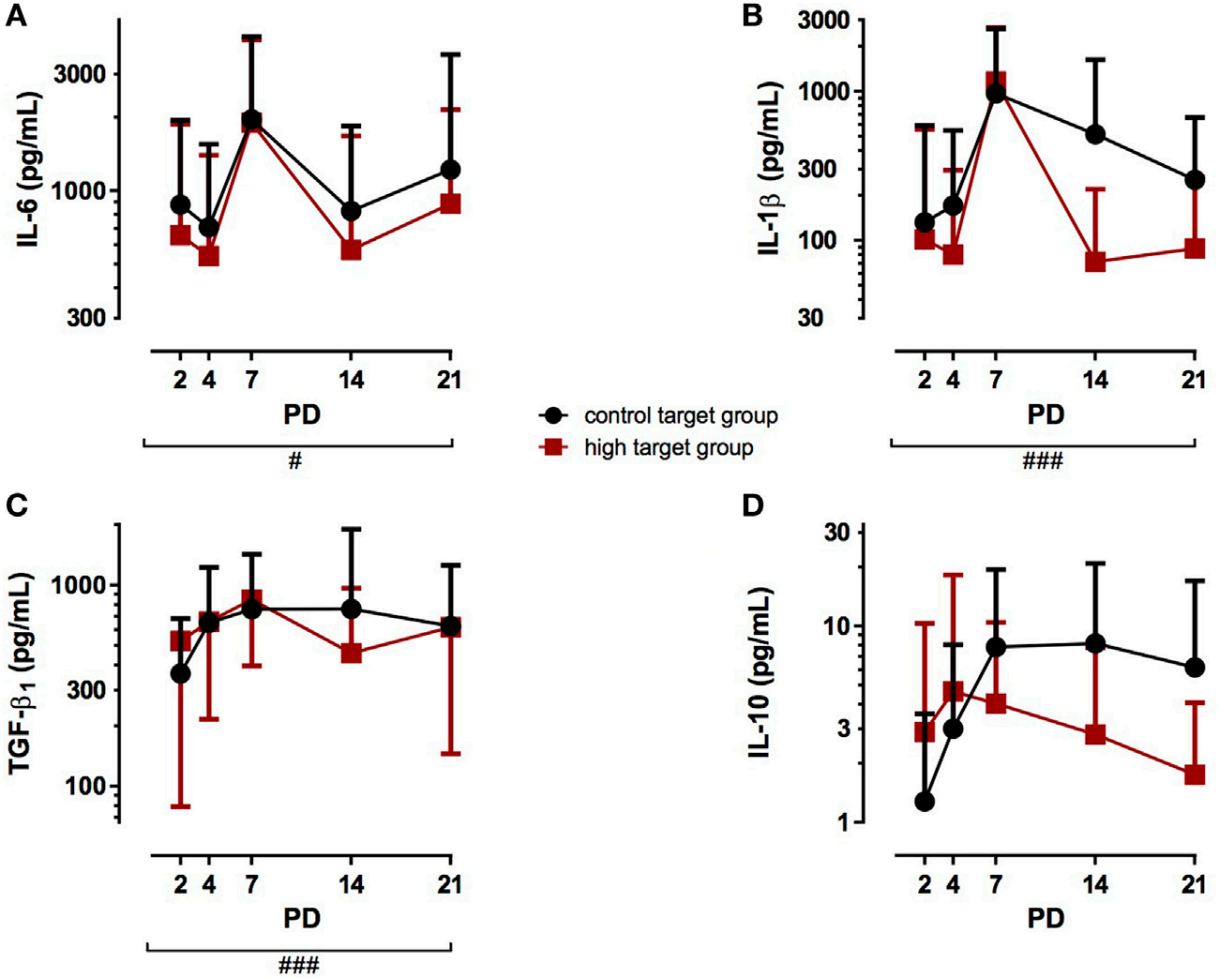
Cytokine tracheal aspirates (TA) concentrations in infants treated with high PCO_2_ target levels compared to control target levels. Serial TA samples were obtained from PD2 to PD21. Data are displayed as the mean of TA levels and SD on a logarithmic scale. **(A)** Interleukin (IL)-6: number (*n*) of analyzed TA for each postnatal day and group (high PCO_2_ target group/control target group): PD2 *n* = 27/25, PD4 *n* = 24/28, PD7 *n* = 17/17, PD14 *n* = 11/15, PD21 *n* = 11/11. IL-6 TA levels were not affected in the high PCO_2_ target group from PD2 to PD21, but increased with advancing postnatal age in both groups (^#^*p* < 0.05). **(B)** IL-1β: number (*n*) of analyzed TA for each postnatal day and group (high PCO_2_ target group/control target group): PD2 *n* = 27/25, PD4 *n* = 24/28, PD7 *n* = 17/17, PD14 *n* = 11/15, PD21 *n* = 11/11. IL-1β TA levels strongly increased with advancing postnatal age in both groups (^###^*p* < 0.001). High PCO_2_ target levels did not affect IL-1β TA levels from PD2 to PD21. **(C)** Transforming growth factor (TGF)-β_1_: number (*n*) of analyzed TA for each postnatal day and group (high PCO_2_ target group/control target group): PD2 *n* = 26/25, PD4 *n* = 23/27, PD7 *n* = 15/17, PD14 *n* = 11/15, PD21 *n* = 11/11. TGF-β_1_ TA levels were not altered by the treatment group from PD2 to PD21, but demonstrated elevated TA levels with advancing postnatal age in both groups (^###^*p* < 0.001). **(D)** IL-10: number (*n*) of analyzed TA for each postnatal day and group (high PCO_2_ target group/control target group): PD2 *n* = 27/25, PD4 *n* = 24/28, PD7 *n* = 17/17, PD14 *n* = 11/15, PD21 *n* = 11/11. IL-10 TA levels were not affected by the target group or postnatal age. PD, postnatal day.

Postnatal age also affected IL-6 TA concentration since levels of both, the high target group and the control target group, significantly increased over the study period from PD 2 to PD 21 (Figure 2A; *p* < 0.05). The different PCO_2_ target levels, however, did not affect IL-6 TA levels from PD 2 to PD 21. Similarly, IL-1β TA levels were not affected by the PCO_2_ target group (Figure 2B), but postnatal age strongly increased IL-1β TA levels in both groups from PD 2 to PD 21 (*p* < 0.001). Furthermore, TGF-β_1_ TA levels increased with advancing postnatal age (Figure 2C; *p* < 0.001), but no difference in TGF-β_1_ TA levels was observed between the high target group and the control target group. IL-10 TA levels were neither affected by the PCO_2_ target group nor postnatal age, as no significant differences were detected during the study period (Figure 2D). In addition, different PCO_2_ target levels did not affect NPY TA levels, while postnatal age decreased NPY TA levels over the study period from PD 2 to PD 21 (Table 2; *p* < 0.05).

**Figure 3:**
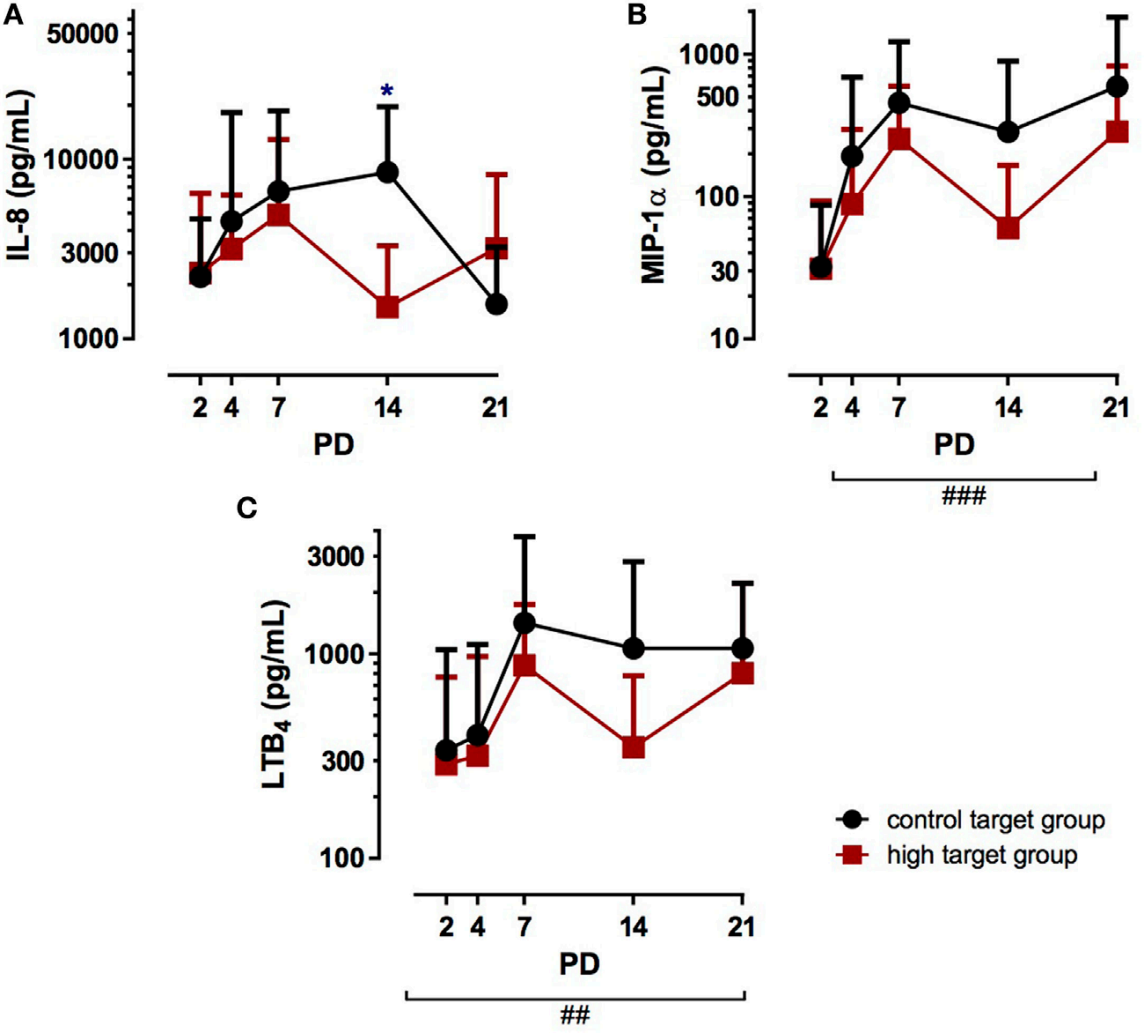
Chemokine tracheal aspirates (TA) concentrations in infants treated with high PCO_2_ target levels compared to control target levels. Serial TA samples were obtained from PD2 to PD21. Data are displayed as the mean of TA levels and SD on a logarithmic scale. **(A)** Interleukin (IL)-8: number (*n*) of analyzed TA for each postnatal day and group (high PCO_2_ target group/control target group): PD2 *n* = 25/25, PD4 *n* = 28/28, PD7 *n* = 17/17, PD14 *n* = 15/15, PD21 *n* = 11/11. IL-8 TA levels were not affected in the high PCO_2_ target group from PD2 to PD7, but on PD14, high PCO_2_ target levels significantly decreased IL-8 TA levels (^↔^*p* < 0.05). On PD21, no difference was observed between the target groups and postnatal age did not affect IL-8 TA levels. **(B)** Macrophage inflammatory protein (MIP)-1α: number (*n*) of analyzed TA for each postnatal day and group (high PCO_2_ target group/control target group): PD2 *n* = 27/25, PD4 *n* = 24/28, PD7 *n* = 17/17, PD14 *n* = 11/15, PD21 *n* = 11/11. MIP-1α TA levels were not altered by the treatment from PD2 to PD21, but demonstrated elevated TA levels with advancing postnatal age in both groups (^###^*p* < 0.001). **(C)** Leukotriene B_4_ (LTB_4_) TA levels were not affected by the target group, but demonstrated elevated TA levels with advancing postnatal age in both groups (^##^*p* < 0.01). PD, postnatal day.

No differences in IL-8 TA levels between the target groups were observed on PD 2 to PD 7 (Figure 3A), but on PD 14, the high target group showed significantly lower IL-8 TA levels with 1.499 ± 1.792 pg/ml compared to 8.480 ± 11.107 pg/ml in the control target group (*p* < 0.05). However, the subsequent measurement on PD 21 showed no differences of IL-8 TA levels between both target groups. In addition, postnatal age did not affect IL-8 TA levels. In contrast to IL-8, postnatal age strongly affected MIP-1α TA levels, which significantly increased from PD 2 to PD 21 in both target groups (Figure 3B; *p* < 0.001). The PCO_2_ target group did not significantly alter MIP-1α TA levels, as no difference was observed between the groups. Similarly, LTB_4_ TA levels were not affected by PCO_2_ target levels from PD 2 to PD 21 (Figure 3C). However, postnatal age strongly increased LTB_4_ TA levels in both groups (*p* < 0.01).

**Figure 4:**
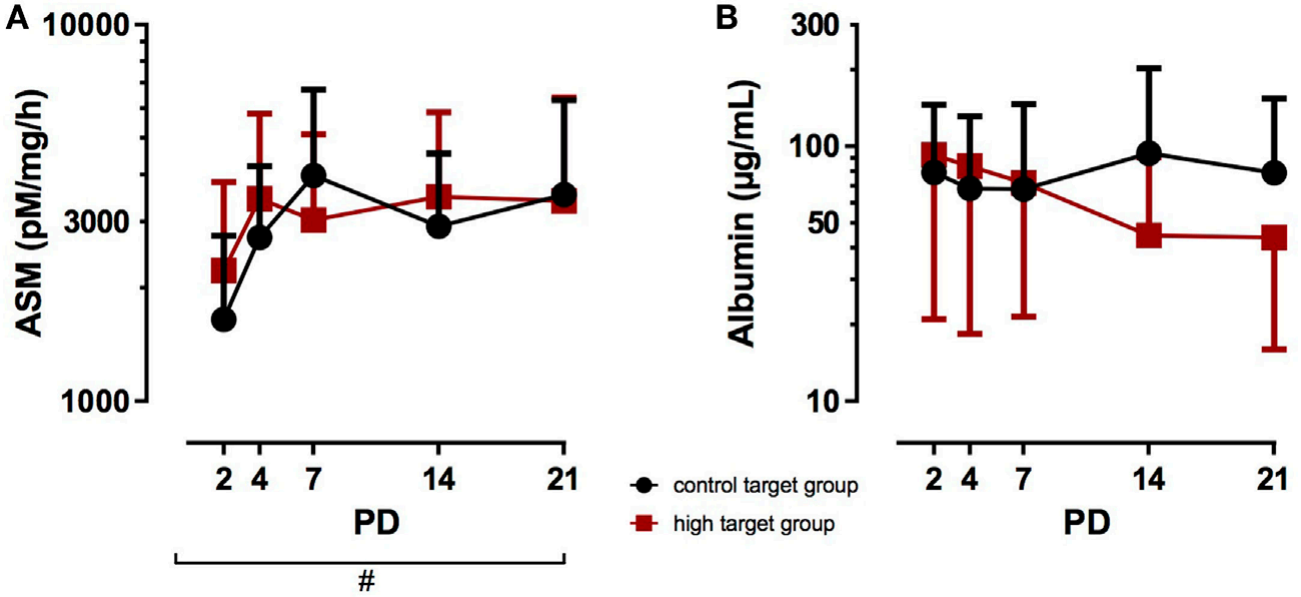
Acid sphingomyelinase (ASM) and albumin tracheal aspirates (TA) concentrations in infants treated with high PCO_2_ target levels compared to control target levels. Serial TA samples were obtained from PD2 to PD21. Data are displayed as the mean of TA levels and SD on a logarithmic scale. **(A)** ASM: number (*n*) of analyzed TA for each postnatal day and group (high PCO_2_ target group/control target group): PD2 *n* = 27/25, PD4 *n* = 24/28, PD7 *n* = 17/17, PD14 *n* = 11/15, PD21 *n* = 11/11. ASM TA levels were not affected in the high PCO_2_ target group from PD2 to PD21, but increased with advancing postnatal age in both groups (^#^*p* < 0.05). **(B)** Albumin: number (*n*) of analyzed TA for each postnatal day and group (high PCO_2_ target group/control target group): PD2 *n* = 27/25, PD4 *n* = 24/28, PD7 *n* = 17/17, PD14 *n* = 11/15, PD21 *n* = 11/11. Albumin TA levels were not affected by the target group or postnatal age. PD, postnatal day.

Finally, the PCO_2_ target levels did not alter the ASM TA levels, as no significant difference was observed between the high target group and the control target group from PD2 to PD21 (Figure 4A), while postnatal age significantly increased ASM TA levels in both target groups (*p* < 0.05). Albumin TA levels were not affected by postnatal age or the target group from PD 2 to PD 21 (Figure 4B).

**Figure 5:**
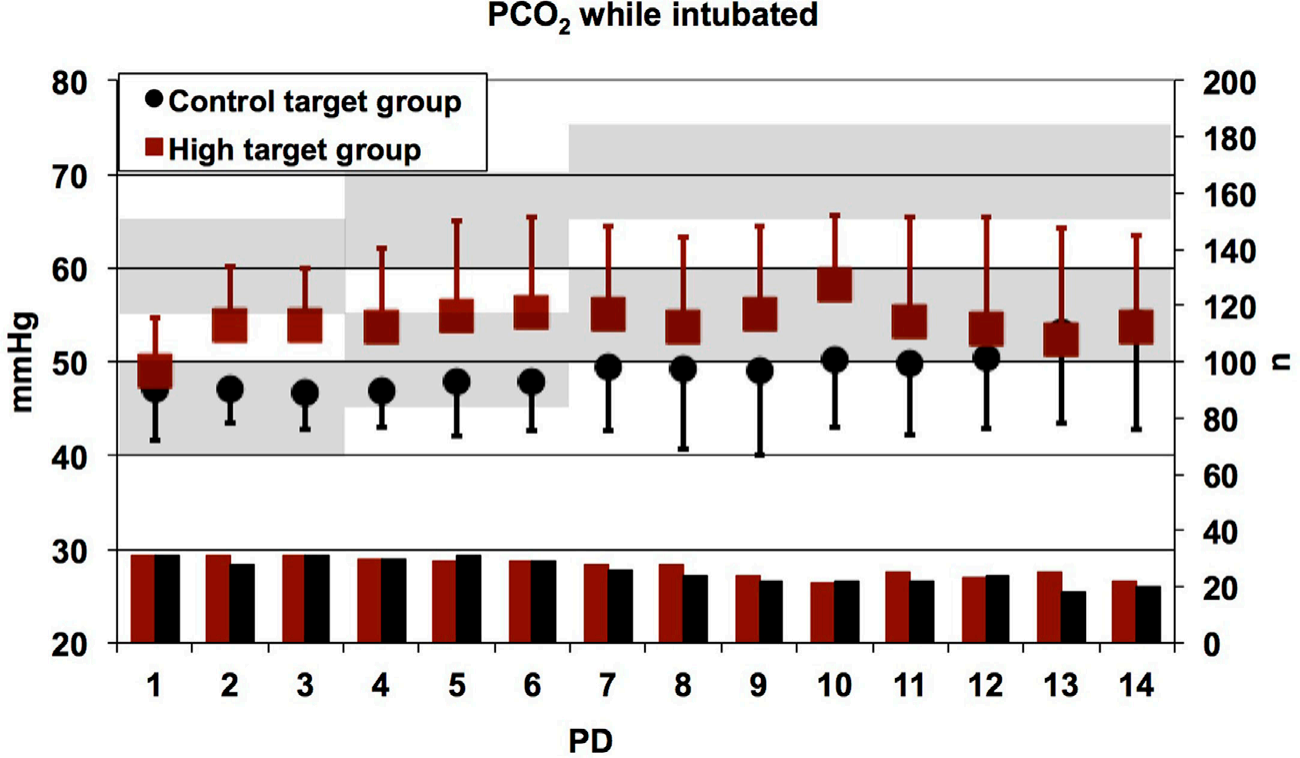
Daily mean values of the partial pressure of carbon dioxide (PCO_2_) in all patients who were intubated at the time of measurement. Error bars indicate SDs; lower bars indicate numbers of patients contributing data. Shaded areas indicate the target ranges of the high target and control target groups. The PCO_2_ values were significantly higher in patients randomized to the high target group as compared to the control target group (linear mixed effects regression model, *p* < 0.0001), although the high target range was frequently not achieved owing to the patients’ own respiratory efforts. The main reason for absent data was extubation. PD, postnatal day.

**Figure 6:**
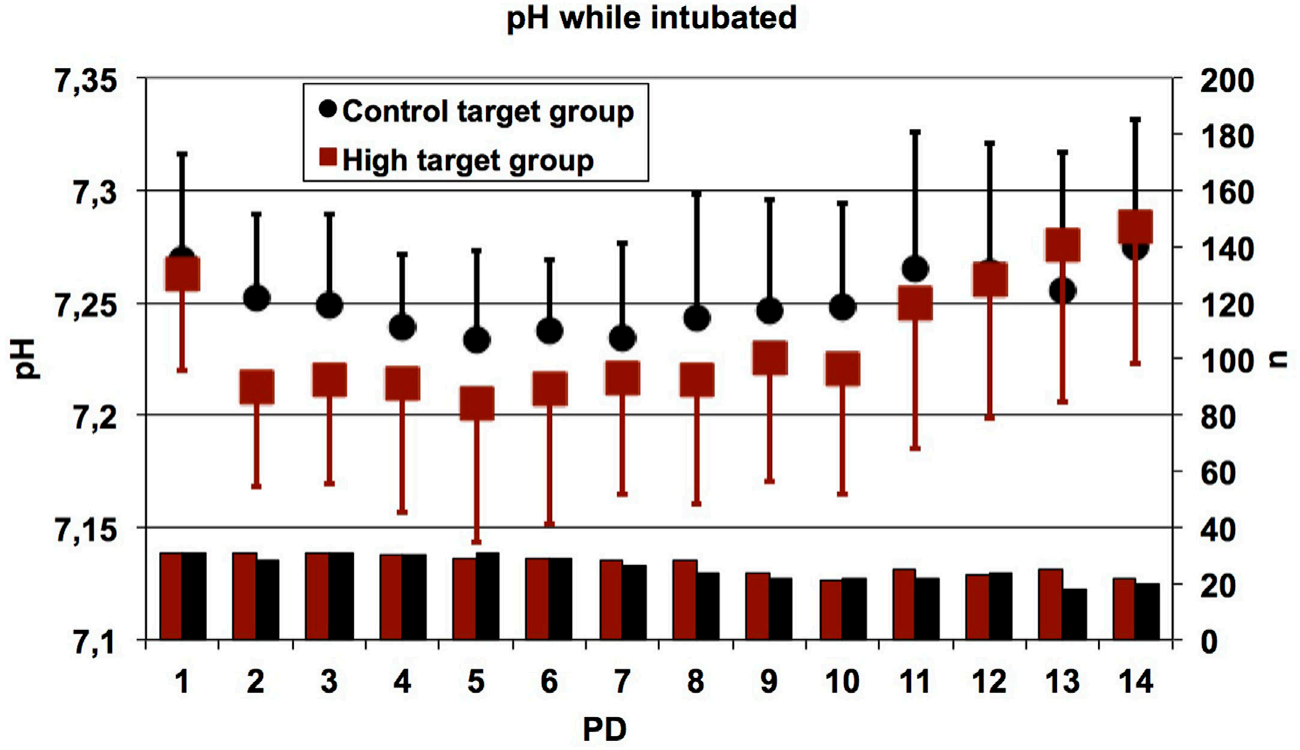
Daily mean values of the pH in all patients who were intubated at the time of measurement. Error bars indicate SDs; lower bars indicate numbers of patients contributing data. The pH values were significantly lower in patients randomized to the high target group as compared to the control target group (linear mixed effects regression model, *p* < 0.0001). The main reason for absent data was extubation. PD, postnatal day.

Day-by-day mean values of PCO_2_ (high PCO_2_ target group: 54.07 ± 8.36 mmHg and control target group 48.94 ± 7.05 mmHg) and pH (high PCO_2_ target group: 7.23 ± 0.06 and control target group 7.25 ± 0.04) differed significantly between study groups, as intended in the study protocol (Figures 5 and 6). In summary, no pronounced differences in TA mediator levels of preterm infants between the high PCO_2_ target group and the control target group from PD 2 to PD 21 were observed.

**Figure 7:**
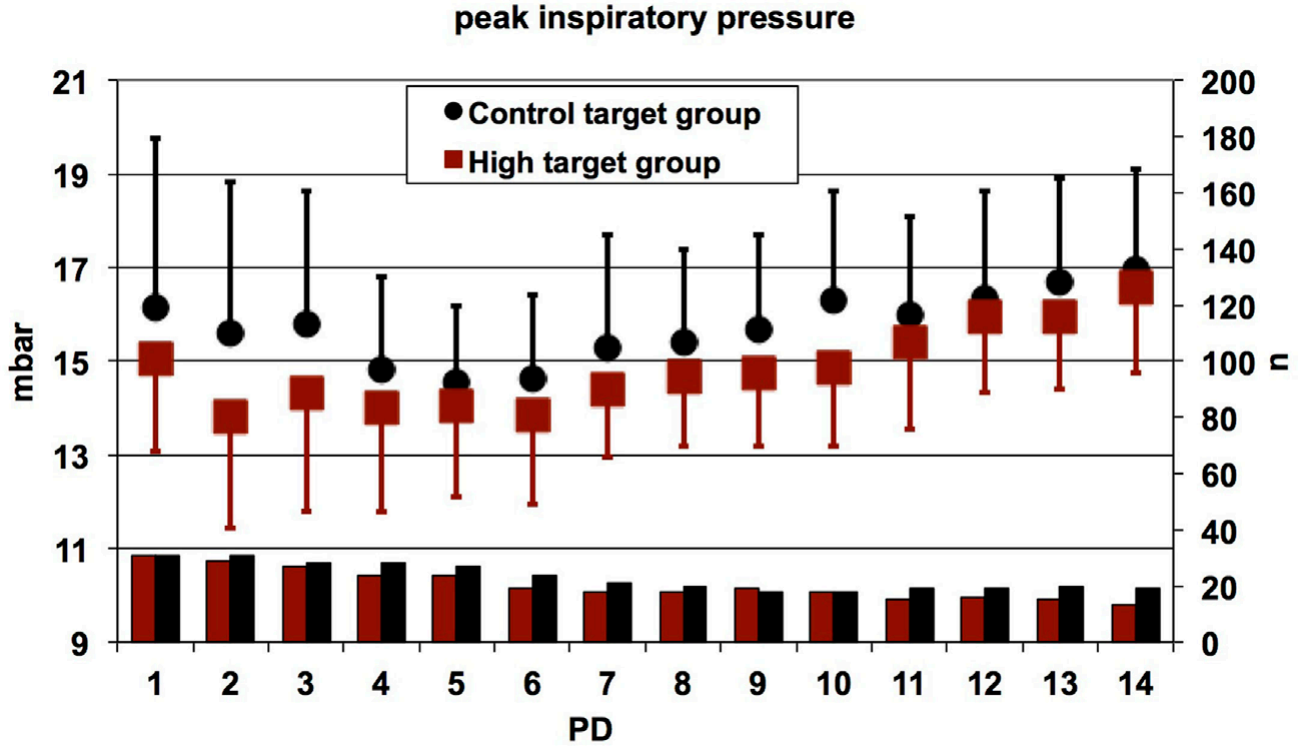
Daily mean values for the peak inspiratory pressure (PIP) in conventionally ventilated patients. Error bars indicate SDs; lower bars indicate numbers of patients contributing data. The PIP values were significantly lower in patients randomized to the high target group as compared to the control target group (linear mixed effects regression model, *p* = 0.01). The main reason for absent data was the use of high-frequency oscillatory ventilation or proportional assist ventilation or extubation. PD, postnatal day.

Daily mean values for the PIP were significantly lower in patients randomized to the high target group as compared to the control target group (linear mixed effects regression model, *p* = 0.01), suggesting increased weaning efforts in the high target group (Figure 7).

## Discussion

In contrast to our initial hypothesis, high PCO_2_ target levels did not result in lower inflammatory activity concerning the analyzed factors IL-6, IL-1β, LTB_4_, TGF-β_1_, NPY, MIP-1α, albumin, and ASM. Nitrite TA levels were reduced in the high target group on PD 21. Since PCO_2_ targets were discontinued on PD 14 according to the study protocol, a difference observed only on PD 21 is not convincing. The anti-inflammatory mediator IL-10 was also not affected by high PCO_2_ target levels. Only IL-8 showed a significantly reduced TA level on PD 14 in the high target group, which did not persist until the next measured time point, PD 21.

Overall, the data demonstrate that inflammatory mediators in TA of preterm infants are not affected by different PCO_2_ targets as used in this trial, which has not been addressed before. These results are consistent with the results of the PHELBI multicenter trial showing neither a reduction of BPD incidence nor BPD severity in the high target group and may thus help to explain the clinical results of the PHELBI trial (42). Since inflammatory processes are a main factor in the pathogenesis of BPD (21), we assume that the comparable levels of inflammatory mediators observed in our study indicate that inflammatory processes were of similar strength in both study groups leading to an equal incidence and severity of BPD.

Inflammatory mediators are thought to have various effects on lung tissue and contribute to lung pathology. Elevated TA cytokine levels indicate pathologic immune responses leading to inflammatory processes in the lung. Different studies suggest that IL-1β, IL-6, IL-8, MIP-1α, LTB_4_, and TGF-β_1_ levels are associated with subsequent BPD development and promote lung fibrosis (24, 27–29, 48–52). Notably, TGF-β_1_ was demonstrated to inhibit alveolar fluid clearance by downregulating β_2_-adrenergic receptors (52, 53). In addition, pro-fibrotic TGF-β_1_ stimulates collagen synthesis and epithelial–mesenchymal transition, contributing to interstitial thickening *in vivo* (54). In contrast to our results, mouse pups exposed to chronic hypercapnia exhibit an altered lung matrix composition with decreased collagen levels (55), suggesting beneficial anti-fibrotic effects of high PCO_2_ levels. However, we did not observe an effect of high PCO_2_ levels on TGF-β_1_ TA concentrations in preterm infants. Furthermore, IL-8 and LTB_4_ (24, 56), as well as IL-6 and NPY (23, 29, 57, 58) contribute to lung edema by increasing microvascular permeability *in vitro*. Elevated microvascular permeability is commonly associated with increased albumin concentrations in TA (24). We neither observed effects on LTB_4_, IL-6, and NPY induced by moderate permissive hypercapnia nor on albumin concentrations, suggesting no difference in microvascular permeability between the study groups. In contrast to our results, hypercapnia reduced IL-6 and IL-1β expression in murine lung tissue of a paraquat-induced acute lung injury model accompanied by decreased numbers of neutrophils in lung tissue and inflammatory infiltration in alveolar septa compared to normoxia (59). However, results of this study were obtained after 1 h of mechanical ventilation and preclude assessment of long-term effects. Although reduced TA levels of IL-8 were detected in the high PCO_2_ target group on PD 14, the reduction of IL-8 levels did not persist until PD 21, questioning the physiological relevance of the observed effect. Another parameter contributing to lung edema is platelet-activating factor (PAF), which acts by activating the cyclooxygenase pathway and ASM. More recently, ceramide and ASM, the enzyme synthesizing ceramide, were shown to be involved in lung edema formation induced by PAF (37, 44). Furthermore, ceramide was shown to induce apoptosis in lung epithelial cells (35, 36). There has not yet been a study testing the association between ASM levels and the development of BPD. Taken together, levels of inflammatory and fibrotic mediators were similar in both study groups.

Although we speculated that high PCO_2_ target levels might reduce mechanical stress, PCO_2_ target levels did not seem to influence pulmonary inflammatory activity. This might be due to altered pH values, which possibly affects a large number of enzymes that are out of their pH optimum, thus counteracting any benefits. In contrast to our results, adult patients suffering from acute respiratory distress syndrome demonstrated beneficial reductions of pulmonary inflammatory cytokines and neutrophils, which was achieved by lowering the intensity of mechanical ventilation and thus tolerating higher PCO_2_ (60). The reason for the difference of effects of high PCO_2_ target levels between adults and preterm infants remains to be determined.

Hypercapnia in adult and newborn rodent models demonstrated attenuation of lung neutrophil recruitment, pulmonary cytokine concentrations, cell apoptosis, and oxygen-derived and nitrogen-derived free radical injury (61). Neonatal rats exposed to 60% oxygen for 14 days showed phagocyte influx, interstitial thickening, and impaired alveolar formation, which was attenuated by concurrent hypercapnia (5.5% CO_2_) (62). Thus, inhaled 5.5% CO_2_ provided partial protection against parenchymal and vascular injury in a mouse model of chronic neonatal lung injury, although the authors acknowledge possible critical differences between permissive and therapeutic hypercapnia in their effects on the lung (62). More recent studies suggested that hypercapnia might also have undesirable effects on lung tissues (63–66). Hypercapnic acidosis impairs pulmonary epithelial wound healing (67), which is NF-κB dependent and involves inhibition of cellular migration. Thus, hypercapnic acidosis might attenuate injury pathways, but on the other hand, it possibly interferes with lung repair (68). Moreover, lung fluid clearance is impaired by hypercapnia independently of pH by triggering endocytosis and thus inhibition of Na,K-ATPase activity in alveolar epithelial cells (69). Another study demonstrated an association between higher PCO_2_ levels during the first few days of life and the subsequent incidence of BPD (13), further questioning the potential clinical benefit of hypercapnia in preterm infants.

Postnatal age of the infants affected almost every analyzed factor demonstrated by significant changes of TA nitrite, IL-6, IL1β, TGF-β_1_, MIP-1α, NPY, LTB_4_, and ASM levels. The inflammatory mediators IL-6, IL1β, TGF-β_1_, MIP-1α, LTB_4_, and ASM increased with advancing postnatal age, while nitrite and NPY levels declined during the study period.

Small differences in TA cytokine levels may be missed in our study because of the small sample size. However, we limited the collection of TA to the largest center of all participating centers of the PHELBI trial to counteract inter-center variations such as differences of tracheal suctioning procedures and clinical care. Therefore, even a multicenter TA sampling study may not yield more precise results and more statistically significant differences. Furthermore, if there are clinically important differences in a larger sample size, unequivocal trends should already be observed in analyses of inflammatory mediators alike. Thus, we assume that pulmonary inflammatory activity was indeed similar in both study groups and important differences would not have become detectable with a larger sample size. Moreover, the biochemical results go along with the clinical outcome in this study, which indicates that high target levels were as beneficial as control target levels in terms of ventilator-induced lung injury, lung inflammation, and the development of chronic lung disease (42). In addition, our results agree with the clinical results of other randomized controlled studies, which also did not observe a reduced incidence of BPD associated with permissive hypercapnia in preterm infants (16, 32, 70).

According to the definition, 12 of 62 infants suffered from BPD in our study, which were distributed statistical comparably in both treatment groups. Thus, similar cytokine levels in both groups were followed by a similar clinical outcome in the patients studied here as well as in the main multicenter trial (42), which might be viewed as a prediction of the main trial outcome by the observed cytokine levels. Differences in the rate of BPD in comparison to previous studies (24, 25, 28, 71) might be explained by different BPD definitions and the multitude of improvements that have been introduced into neonatal care.

A limitation of this study is the declining number of infants supplying TA with advancing postnatal age, because infants are extubated as soon as clinically possible to limit potential lung damage from mechanical ventilation. Therefore, infants, who were more prone to develop BPD, remained longer in the study and supply more TA due to continuing invasive mechanical ventilation. Thus, after 14 or 21 days of life, the acquired TA may not be completely representative for the whole group, since TA was only available from infants who still received invasive mechanical ventilation at that time. Furthermore, normalization of TA to correct for dilution is still controversial. Different techniques have been proposed, including albumin content, urea, or secretory immunoglobulin A concentrations. However, no uniformly accepted correction factor is currently available. We did not correct our results for dilution and expressed the data per milliliter of TA, as recommended by the European Respiratory Task Force on Bronchoalveolar Lavage in children (45, 46).

## Conclusion

No differences in the levels of most cytokines were found when comparing infants with control or high PCO_2_ targets. In addition, there were no differences in the clinical outcome, which was in accordance with the results of the main multicenter PHELBI trial. We assume that higher PCO_2_ target ranges are as beneficial as the control target range. Indeed, high target levels may reduce mechanical stress for the pulmonary parenchyma, but possible suboptimal pH-values might impede enzymes, which may be further inhibited directly by high PCO_2_ concentrations. Overall, the positive effects of high target levels such as less bronchoalveolar damage may be abrogated by the negative effects. This corresponds to the results of the TA measurements.

## Ethics Statement

This study was carried out in accordance with the recommendations of the International Conference of Harmonization Good Clinical Practice Guidelines and the protocol approved by the institutional review board of the university of Ulm medical faculty. All subjects gave written informed consent in accordance with the Declaration of Helsinki.

## Author Contributions

SG: substantial contribution to acquisition of data, analysis and interpretation of data, as well as drafting the article and giving final approval to be submitted. ML: participated in revising the article critically for important intellectual content and has given final approval of the version to be submitted. UU, YY, and SU: substantial contribution to analysis of tracheal aspirates. HF and HH: made substantial contribution to conception and design of the trial. JD: substantial contribution to statistical analysis of data. UT: substantial contribution to acquisition of data, analysis and interpretation of data, as well as revising the article critically for important intellectual content and giving final approval to be submitted.

## Conflict of Interest Statement

The authors declare that the research was conducted in the absence of any commercial or financial relationships that could be construed as a potential conflict of interest.

## Abbreviations

ASM, acid sphingomyelinase; BPD, bronchopulmonary dysplasia (chronic lung disease of prematurity); CPAP, continuous positive airway pressure; ELISA, enzyme-linked immunosorbent assay; IL-1, interleukin-1; IL-6, interleukin-6; IL-8, interleukin-8 (also called CXCL-8); IL-10, interleukin-10; IVH, intraventricular hemorrhage; LTB_4_, leukotriene B_4_; MIP-1α, macrophage inflammatory protein 1 alpha; NPY, neuropeptide Y; PCO_2_, partial pressure of carbon dioxide; PD, postnatal day; PHELBI, permissive hypercapnia in extremely low birth weight infants; TA, tracheal aspirate; TGF-β_1_, transforming growth factor beta-1.
